# *Falcaria vulgaris* leaves extract as an eco-friendly corrosion inhibitor for mild steel in hydrochloric acid media

**DOI:** 10.1038/s41598-023-30571-6

**Published:** 2023-03-06

**Authors:** Mohammadreza Alimohammadi, Mohammad Ghaderi, Ahmad Ramazani S.A., Mohammad Mahdavian

**Affiliations:** 1 Chemical and Petroleum Engineering Department, Sharif University of Technology, Tehran, Iran; 2 Surface Coating and Corrosion Department, Institute for Color Science and Technology, Tehran, Iran

**Keywords:** Corrosion, Pollution remediation, Surface spectroscopy

## Abstract

Undoubtedly, metal corrosion is one of the most challenging problems faced by industries. Introducing corrosion inhibitors is a reasonable approach to protecting the metal surface. Due to environmental concerns and the toxicity of industrial organic corrosion inhibitors, researchers are continually exploring acceptable replacements. The current study focused on the application of Falcaria Vulgaris (FV) leaves extract to mitigate mild steel (MS) corrosion in a 1 M HCl environment. The polarization findings demonstrated that the corrosion current density decreased from 264.0 µA/cm^2^ (for the sample submerged in the blank solution) to 20.4 µA/cm^2^ when the optimal concentration of 800 ppm of FV leaves extract was added to the acid solution. Electrochemical impedance spectroscopy (EIS) analysis revealed an inhibition efficiency of 91.3% at this concentration after 6 h of immersion. It was determined by analyzing several adsorption isotherms that this corrosion inhibitor obeys the Frumkin isotherm. AFM, FE-SEM, and GIXRD surface analyses also supported the findings that adding FV leaves extract can reduce metal damage by adsorption on the metal surface.

## Introduction

Nowadays, metal corrosion is one of the most severe challenges confronting industries. Mild steel (MS), one of the most ubiquitous building materials, is highly susceptible to corrosive ions despite its remarkable qualities, including excellent mechanical capabilities and affordability^1^. Various methods are used to remove contaminants and rust from the MS surface, the most common of which is acid washing (especially using HCl)^2^. Consequently, it is crucial to employ strategies that minimize the rate of metal dissolution. Anti-corrosion coatings, corrosion inhibitors, anodic and cathodic protection, and other approaches have been proposed for this goal^3^. Among all these methods, corrosion inhibitors stand out as a promising approach. In order to select the proper corrosion inhibitor, three crucial factors must be taken into account: (1) effective adsorption, and competence in safeguarding the metal surface, (2) environmentally friendliness, and (3) affordability.

Generally, corrosion inhibitors containing heteroatoms (such as sulfur, oxygen, nitrogen, and phosphorus) can form a bond with the iron's vacant d orbital via their non-bonding electron pair, preventing metal corrosion by producing a protective layer^4,5^. Furthermore, compounds comprising aromatic rings and polar groups (such as C=O, –NH_2_, –OH, etc.) can be readily adsorbed on the metal surface via electrostatic attraction^6,7^. Even though some industrial organic corrosion inhibitors have the property mentioned above and exhibit potent inhibition against harsh ions, they suffer from the absence of two other qualities (they might be toxic and expensive)^8–11^. Hence, it is vital to discover an alternative with all the desirable features.

Recently, green organic corrosion inhibitors, including plant extracts^12^, expired drugs^13^, and ionic liquids^14^ with effective compounds, have been introduced as a substitution for toxic convectional corrosion inhibitors. Plant extracts, comprising leaf^15^, fruit ^16^, and seed ^17^ extracts, are generally biocompatible, biodegradable, and cost-effective. Also, donor electron components such as aromatic groups, heteroatoms, and compounds with π electrons in plant extract can further confirm their potential to be used as potent corrosion inhibitors^18–21^.

Many efforts have shown the role of different plant extracts in the protection of MS surfaces in acidic media. Mostafatabar et al. evaluated the inhibitory effect of carrot pomace extract. They clarified that the extract molecules could be adsorbed physicochemically and generate a protective layer on the MS panel, leading to 95% efficiency at 400 ppm extract concentration according to polarization assessment^22^. In another report, garcinia cambogia fruit rind extract derived from aqueous and alcoholic media was introduced as a green corrosion inhibitor demonstrating mix mode (cathodic and anodic) protection via Langmuir and Temkin adsorption isotherm, respectively^23^. Dehghani et al. investigated the inhibition action of the rosemary extract at different concentrations and temperatures. Their results showed that increasing the rosemary extract concentration to 800 ppm enhanced the corrosion inhibition efficiency to 92.0%^24^. Moreover, exploration of the inhibition performance of other plant extracts, including Mish Gush^25^, *Xanthium strumarium*^26^, *Eriobotrya japonic*a Lindl^27^, *Cardaria draba*^28^, *Urtica dioica*^29^, *Arbutus unedo* L^30^, *Euphorbia heterophylla* L^31^ and *Thymus vulgaris*^32^, Onion–garlic^33^ obviously endorsed the potential of plant extracts as corrosion inhibitors.

**Figure 1:**
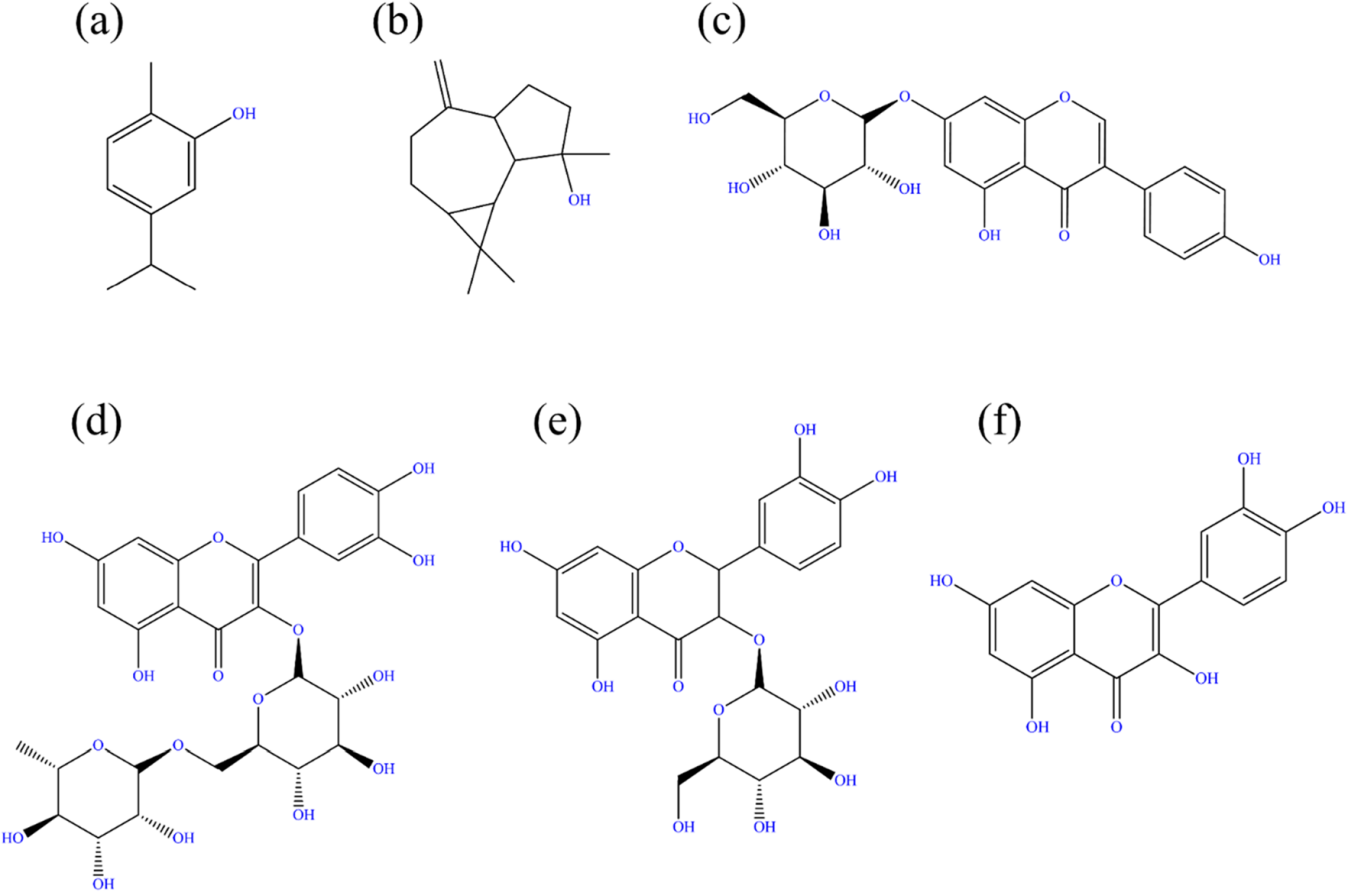
The chemical structure of Carvacrol (**a**), Spathulenol (**b**), Genistin (**c**), Rutin (**d**), Quercetin-3-*O*-glucoside (**e**), and Quercetin (**f**).

*Falcaria vulgaris* (FV) is a species of the Apiaceae family found in West Asia, Europe, and the United States. This plant has been used for medical applications such as healing skin and gastrointestinal diseases in many regions of Iran. Furthermore, the antibacterial and antioxidant properties of FV have been approved due to the existence of carvacrol and spathulenol in its structure. In addition to the mentioned compounds, FV leaves extract contains genistin, rutin, quercetin-3-*O*-glucoside, and quercetin (Fig. 1)^34,35^.

In current study, we aimed to investigate the inhibitory performance of FV extract towards corrosion mitigation of MS submerged in 1 M HCl medium. Electrochemical measurements such as EIS and polarization were used to explore this compound's anti-corrosion properties. In addition, the morphology and topology of the surface were examined by exploiting energy-dispersive X-ray analysis (EDX), field-emission scanning electron microscopy (FE-SEM), and atomic force microscopy (AFM). Also, Fourier-transform infrared spectroscopy (FTIR) and grazing incidence X-ray diffraction (GIXRD) were used to assess the FV-based layer formed on the MS surface.

## Materials and methods

### Extraction process

FV leaves were collected from the Markazi province, Iran (with permission of the landowner) and dried and powdered after washing with distilled water. For extract preparation, 15 g of FV leaves powder was poured into 500 ml of deionized water and agitated with a heater stirrer for 12 h at 70 °C. After that, filtration was performed using filtering paper to separate dark brown liquid and solid, and in the second step, the obtained solution was centrifuged at 4000 rpm for 5 min. Finally, the prepared extract was dried at 45 °C overnight. All methods were performed in accordance with the relevant guidelines and regulations. The main chemical structure of FV leaves extracts is illustrated in Fig. 1, as already mentioned.

### Sample preparation

MS plates (ST12, Foolad-E-Mobarakeh Co., Iran) were used as a working electrode. Hydrochloric acid (37%, Doctor-Mojalali Co., Iran) was diluted with distilled water to prepare 1 M HCl. Different grades of silicone carbide paper (400–1000) were used to remove the surface scales. After that, the surface was cleaned using industrial-grade of acetone. Finally, different concentrations of FV extract in 1 M HCl (0, 400, 600, 800, and 1000 ppm) were prepared for electrochemical measurement (the solubility of FV leaves extract was higher than 5 g L^−1^).

### Electrochemical measurements

CorrTest (CS350, China) potentiostat instrument was exploited to check the corrosion inhibition effect of FV leaves extract. The electrochemical setup comprised three-electrode, including MS (contact surface = 1 cm^2^), Calomel and Pt rod. EIS analysis was conducted by applying 10 mV AC voltage at open circuit potential (OCP) in the frequency range of 0.01 to 10,000 Hz. Also, the polarization test was performed within the voltage range of −250 to + 250 mV versus OCP with a 0.5 mV/s sweep rate. Three experiments were performed for each concentration to guarantee the reproducibility of data.

### Surface study

To study surface characteristics, MS was soaked in an acidic solution with and without 800 ppm of FV extract for 6 h. After that surface of MS was rinsed with distilled water two times and then dried at room temperature. Morphological and elemental analysis of the MS surface were assessed using FE-SEM (TE-Scan—MIRA3) and EDX (Oxford—X-MAX-80). Also, the surface topology was examined using AFM (Bruker, Icon, United States). The adsorption of FV extract molecules on the MS surface was analyzed using FTIR (Thermo, Avatar, United States), Ultraviolet–visible spectroscopy (UV–Vis, Thermo, Biomate5, United States), and GIXRD (X'Pert PRO MPD PANalytical Company).

## Results and discussion

### Electrochemical examinations

#### OCP

**Figure 2:**
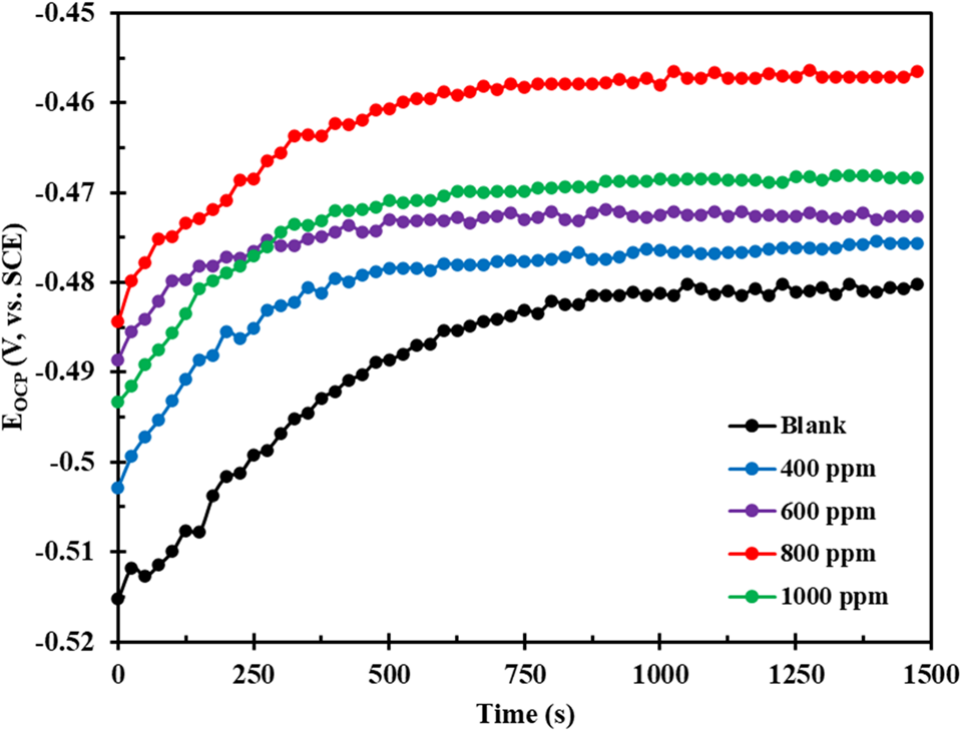
OCP vs time diagrams for MS submerged in an acidic solution without and containing different concentration of FV leaves extract.

The OCP assessment of the MS substrate was conducted during 1500 s immersion in the HCl medium, in the presence and absence of the FV leaves extract. The data depicted in Fig. 2 reveals that the OCP reached a state of equilibrium prior to the completion of the 1500 s, as no discernible deviation from the OCP values was observed after 1000 s. The OCP trend for both conditions was congruent, commencing at a lower potential and progressively augmenting over time, a phenomenon that can be attributed to the formation of an oxide layer on the MS surface as a result of the corrosive species attack until a steady state is attained^36^. Notably, the initial OCP value in the FV-containing solution was higher than that of the blank solution, which may be indicative of the adsorption of inhibitors onto the MS surface^36^. Upon examination of the OCP values in the presence of FV, it was determined that the maximum deviation of OCP was under 85 mV, indicating that the FV extract displays properties of a mixed-type corrosion inhibitor for MS in a 1 M HCl solution^37^. However, a detailed analysis of the data presented in Fig. 2 highlights that FV primarily exerts its inhibition effects through inhibiting anodic reactions as OCP shows a positive shift upon addition of FV extract^38^.

#### EIS

**Figure 3:**
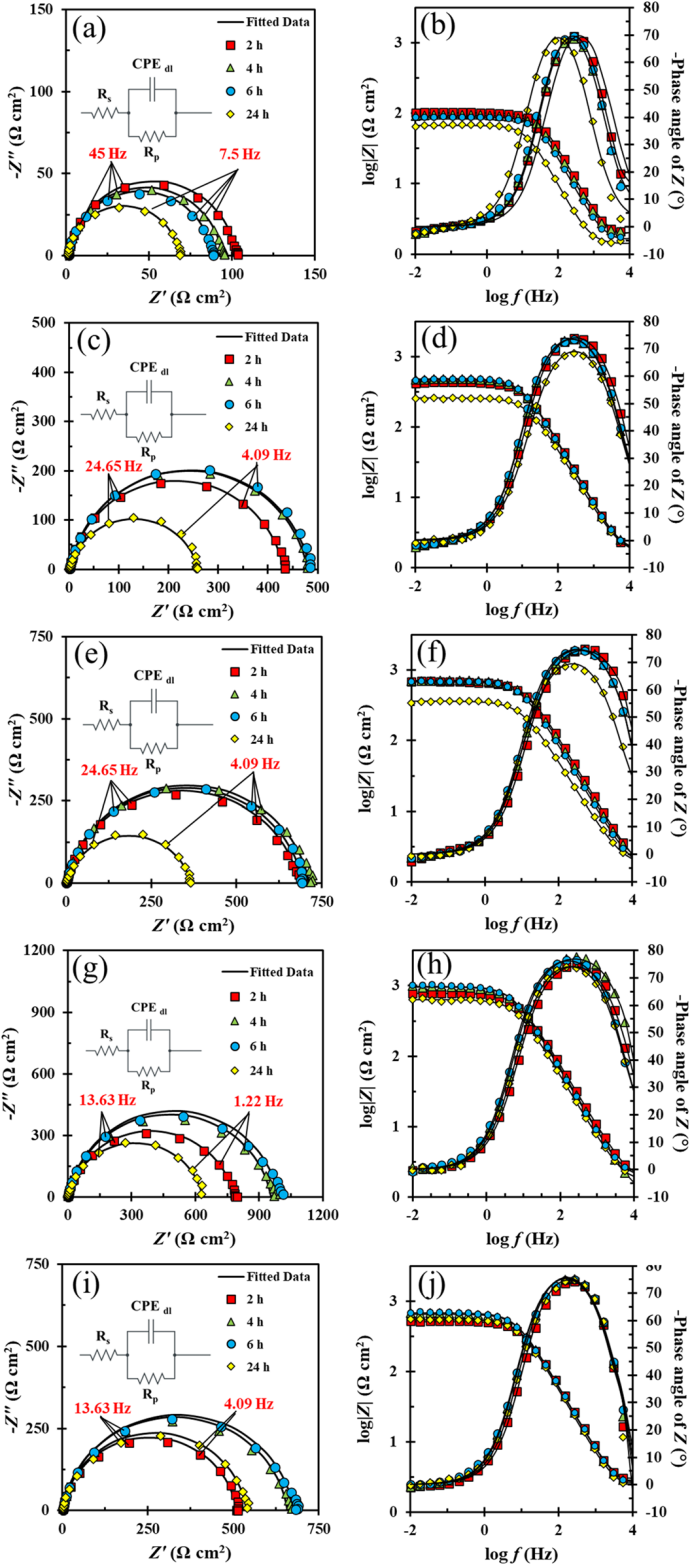
Nyquist (left) and Bode (right) plots of MS soaked in 1 M HCl solution without (**a,b**) and containing 400 (**c,d**), 600 (**e,f**), 800 (**g,h**), and 1000 (**i,j**) ppm of FV extract.

EIS analysis was used to scrutinize the corrosion-inhibiting competence of FV leaves extract. Figure 3 illustrates the Nyquist and Bode plots of MS immersed in acidic electrolytes containing various concentration of FV extract solution (400–1000 ppm) and blank solution. A depressed semicircle, representing the roughness of the tested sample's surface, can be seen in all Nyquist diagrams^39^. It is of note that the Nyquist graph's shape remained unchanged when various inhibitor concentrations were added, indicating that the mechanism of the corrosion reaction remained unaltered. Furthermore, only one time constant is visible on the Bode-phase angle diagram, revealing that the charge transfer mechanism predominates at the metal/electrolyte interface^40^.

The EIS data were fitted by exploiting a one-time constant equivalent electrical circuit (chi-square < 0.005) utilizing ZView software for a more precise examination, and the related parameters are reported in Table 1. In this table, the terms *R*_*s*_, *CPE*_*dl*_, *Y*_0_, and *n* connote the electrolyte resistance, constant phase element, admittance, and power of the CPE, respectively. Furthermore, *R*_*p*_ stands for polarization resistance, calculated by adding the charge transfer and film resistance (*R*_*p*_ = *R*_*ct*_ + *R*_*f*_ ) ^19^. As demonstrated in Fig. 3, the smallest diameter of the Nyquist diagram (which indicates *R*_*p*_) corresponds to the sample immersed in the inhibitor-free solution and displays a declining trend over time. The corrosion reactions instigate as soon as the working electrode is soaked in 1 M HCl medium. As a result, the attack of corrosive ions induces MS dissolution and the degradation of the porous oxide layer, culminating in poor MS resistance in the absence of corrosion inhibitors. In contrast, the diameter of the Nyquist diagram was considerably boosted by introducing diverse concentrations of FV leaves extract in the acidic solution. To gain a better insight into the corrosion-inhibiting properties of FV leaves extract, the inhibition efficiency (*η*) was computed using Eq. (1)^41^:

**Table 1:** Electrochemical parameters attained from fitted EIS data.

Concentration (ppm)	Immersion time (h)	*R*_*s*_^a^ (Ω cm^2^)	*R*_*ct*_^b^ (Ω cm^2^)	*CPE*		*C*_*dl*_^e^ (µF cm^−2^)	log |*Z*|_0.01 Hz_ (Ω cm^2^)	*η* (%)	*τ* (s)	*θ*_*EIS*_
				*Y*_*0*_^c^ (µs^n^ Ω^−1^ cm^−2^)	*n*^d^					
Blank	2	1.8 $\pm$ 0.02	100.4 $\pm$ 1.32	72.7 $\pm$ 1.23	0.94 $\pm$ 0.01	40.6	2.00	–	0.004	–
	4	1.8 $\pm$ 0.02	92.1 $\pm$ 1.21	84.7 $\pm$ 1.45	0.94 $\pm$ 0.01	47.9	1.96	–	0.004	–
	6	1.5 $\pm$ 0.03	86.3 $\pm$ 0.9	99.9 $\pm$ 1.67	0.94 $\pm$ 0.01	57.0	1.94	–	0.004	–
	24	1.4 $\pm$ 0.02	66.3 $\pm$ 1.23	237.2 $\pm$ 2.45	0.95 $\pm$ 0.01	150.9	1.81	–	0.01	–
400	2	1.3 $\pm$ 0.08	429.8 $\pm$ 5.36	52.6 $\pm$ 3.18	0.89 $\pm$ 0.01	15.6	2.62	76.6	0.006	61.5
	4	1.6 $\pm$ 0.04	477.0 $\pm$ 4.67	54.3 $\pm$ 1.88	0.89 $\pm$ 0.01	16.9	2.67	80.7	0.008	64.6
	6	1.5 $\pm$ 0.15	482.5 $\pm$ 8.32	55.7 $\pm$ 2.12	0.89 $\pm$ 0.01	16.9	2.66	82.1	0.008	70.3
	24	1.5 $\pm$ 0.04	257.7 $\pm$ 6.89	83.0 $\pm$ 3.47	0.86 $\pm$ 0.01	18.7	2.4	74.3	0.004	87.6
600	2	1.2 $\pm$ 0.15	682.7 $\pm$ 11.12	31.0 $\pm$ 5.10	0.88 $\pm$ 0.01	7.6	2.84	85.3	0.005	81.2
	4	1.6 $\pm$ 0.13	712.0 $\pm$ 8.78	33.1 $\pm$ 3.66	0.89 $\pm$ 0.01	9.4	2.85	87.0	0.006	80.3
	6	1.4 $\pm$ 0.12	694.6 $\pm$ 9.56	37.8 $\pm$ 3.78	0.89 $\pm$ 0.01	10.6	2.83	87.6	0.007	81.4
	24	1.6 $\pm$ 0.08	364.7 $\pm$ 5.83	78.8 $\pm$ 2.22	0.85 $\pm$ 0.01	17.2	2.53	81.8	0.006	88.6
800	2	1.6 $\pm$ 0.31	778.9 $\pm$ 13.87	45.3 $\pm$ 8.16	0.88 $\pm$ 0.01	12.3	2.89	87.1	0.009	69.7
	4	1.0 $\pm$ 0.13	954.1 $\pm$ 12.12	45.8 $\pm$ 4.12	0.9 $\pm$ 0.01	14.1	3.00	90.3	0.013	70.6
	6	1.7 $\pm$ 0.16	988.1 $\pm$ 12.49	47.4 $\pm$ 3.13	0.89 $\pm$ 0.01	16.2	3.00	91.3	0.016	71.5
	24	1.5 $\pm$ 0.17	633.0 $\pm$ 8.44	63.3 $\pm$ 2.11	0.88 $\pm$ 0.01	19.0	2.80	89.5	0.012	87.4
1000	2	2.5 $\pm$ 0.30	506.7 $\pm$ 16.19	89.8 $\pm$ 5.55	0.91 $\pm$ 0.01	19.0	2.71	80.2	0.009	53.2
	4	2.3 $\pm$ 0.27	654.5 $\pm$ 1.2	77.4 $\pm$ 3.98	0.91 $\pm$ 0.01	18.8	2.82	85.9	0.012	60.6
	6	2.2 $\pm$ 0.21	674.7 $\pm$ 12.91	64.9 $\pm$ 3.45	0.91 $\pm$ 0.01	18.4	2.83	87.8	0.012	67.8
	24	2.2 $\pm$ 0.12	535.4 $\pm$ 9.90	82.8 $\pm$ 2.20	0.92 $\pm$ 0.01	21.4	2.74	87.6	0.01	85.8

^a^Standard deviation changed between 1.5 and 2.1%

^b^Standard deviation changed between 2.6 and 3.2%

^c^Standard deviation changed between 2.2 and 4.7%

^d^Standard deviation changed between 1.9 and 3.5%

^e^Standard deviation changed between 3.1 and 5.4%.

1 $$\eta \%=\left(1-\frac{{R}_{p.0}}{{R}_{p. i}}\right)100\%$$

In this equation, *R*_*p,0*_ and *R*_*p, i*_ denote the polarization resistance of MS in the inhibitor-free and inhibitor-containing solution, respectively. Compared to the blank solution, the sample submerged in the inhibitor-containing solution has a greater *R*_*p*_, as shown in Table 1. It is worth mentioning that as the concentration of the corrosion inhibitor increased, the *R*_*p*_ elevated, reaching a maximum of *R*_*p*_ = 988.1 Ω cm^2^ and *η* % = 91.3% at 800 ppm after 6 h of immersion. This remarkable improvement is due to corrosion inhibitor adsorption on the MS surface, which establishes a protective layer and blocks the access of the corrosive electrolyte to the metal surface. Notably, the *η* has dropped slightly after 24 h of immersion, suggesting the extract's long-term capacity to mitigate corrosion of the MS surface.

The Bode diagram can provide more information regarding the corrosion-inhibition activity of FV leaves extract. According to the literature, rising |*Z*| at the lowest frequency (0.01 Hz) and decreasing the phase angle towards -90° (pure capacitor phase angle) at the highest frequency (10,000 Hz) suggests an increase in corrosion resistance^36^. Table 1 and the Bode graphs show that these two parameters (|*Z*|_0.01 Hz_ and phase angle at 10 kHz) for the coupons dipped in the inhibitor-based solution are greater than the blank one, confirming the development of the FV-based protective film on the working electrode's surface. Further beneficial parameters in corrosion studies are capacitance (*C*_*dl*_) and relaxation time (*τ*), which are expressed by Eqs. (2) and (3), respectively^36,42^, and their values are given in Table 1.

2 $${C}_{dl}={Y}_{0}^\frac{1}{n}\times {(\frac{{R}_{s}\times {R}_{p}}{{R}_{s}+{R}_{p}})}^{\frac{1-n}{n}}= \frac{{\varepsilon }^{0} \varepsilon A}{d}$$

3 $$\tau ={R}_{p}\times {C}_{dl}$$

In Eq. (2), *ε*^*0*^ and ε symbolize the dielectric constants of air and the double layer, while *A* and *d* signify the surface area of MS and the electric double layer thickness, respectively. A close inspection of Table 1 implies that when the inhibitor is added to the acidic solution, *C*_*dl*_ significantly lowered in comparison to the inhibitor-free solution. These findings might be related to the replacement of inhibitor components with water molecules, leading to a drop in *ε* and an increase in *d*, resulting in a decrease in *C*_*dl*_^43^. According to the inferences mentioned above, the decline in *C*_*dl*_ can be linked to a reduction in the contact area between corrosive water molecules and the MS surface. Furthermore, the increase in *τ* of the sample soaked in the inhibitor-based solution might be linked to the inhibitor molecule adsorption on the MS surface, which ultimately resulted in a delay in reaching equilibrium following charge distribution^44^.

Surface coverage (*θ*_*EIS*_), which is given by Eq. (4)^45,46^, is another parameter that may be extracted from EIS data:

4 $${\theta }_{EIS} \%=\left(1-\frac{{C}_{dl. i}}{{C}_{dl. 0}}\right)100\%$$

In this equation, *C*_*dl,0*_ and *C*_*dl,i*_ represent the capacitance of MS in solutions without and with FV leaves extract, respectively. The orientation of corrosion inhibitor molecules on the MS surface (horizontal or vertical) can be assessed by comparing *θ*_*EIS*_ with *η*. When a corrosion inhibitor is adsorbed horizontally on a metal surface, it blocks the access of corrosive ions to the metal surface and leads to an increase in *R*_*p*_ and *η*. Consequently, it is demonstrated that *η* > *θ*_*EIS*_ when the horizontal adsorption of inhibitor molecules occurs on the MS surface^43,47,48^. Conversely, when corrosion inhibitor molecules are vertically oriented on the MS surface, the electric double layer thickness rises and, as a result, *C*_*dl*_ decreases. Meanwhile, *R*_*p*_ is less affected because corrosion inhibitor molecules replace just a limited number of water molecules. Thus, *η* < *θ*_*EIS*_ reflects the vertical adsorption of corrosion inhibitors^43,47,48^.

A careful evaluation of Table 1 discloses that the FV leaves extract molecules (which contain diverse compounds such as carvacrol, spathulenol, genistin, etc.) are horizontally adsorbed on the MS surface during 6 h of immersion (*η* > *θ*_*EIS*_). It is worth noting that some compounds in FV leaves extract may be desorbed from the metal surface after 24 h of immersion. Thus, as shown in Table 1, it is not surprising that the orientation of the remaining molecules on the MS surface has altered and become vertical (*η* < *θ*_*EIS*_) after 24 h of immersion at concentrations of 400 and 600 ppm^49^. Higher concentrations (800 and 1000 ppm) also show a similar trend, and after 6 h, *η* declines and *θ*_*EIS*_ rises. Although *η* is still bigger than *θ*_*EIS*_, the fact that these two values are so close to one another after 24 h compared to other times suggests that the corrosion inhibitors have the propensity to switch from horizontal to vertical orientation. This observation can be interpreted as evidence for either the durability of the protective film generated on the MS surface or the existence of more active FV molecules in the solution, which can quickly and efficiently replace the desorbed molecules﻿.

**Figure 4:**
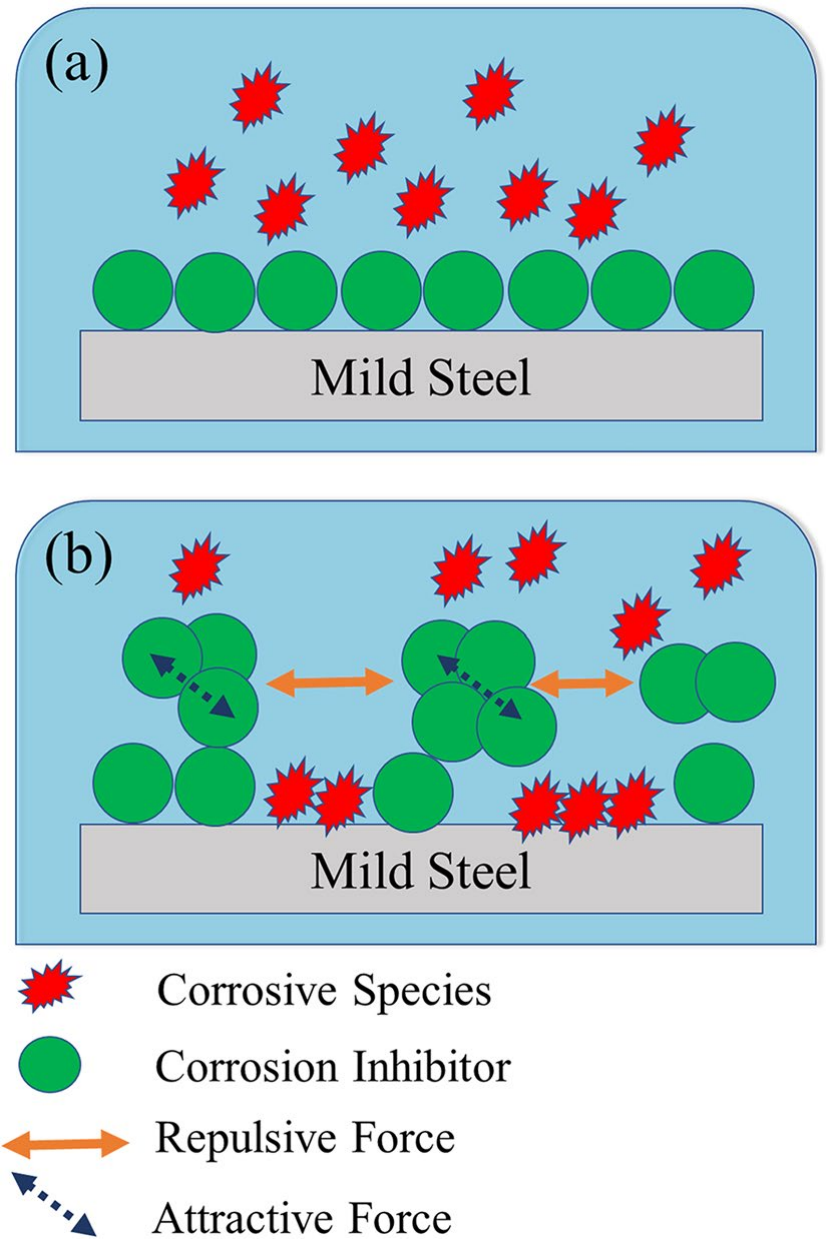
The corrosion inhibitor adsorption process: at optimum concentration (**a**) and above optimum concentration (**b**).

As shown in Table 1, the maximum inhibition efficiency was observed at a concentration of 800 ppm (optimum concentration), whereas raising the concentration to 1000 ppm decreased the polarization resistance and inhibition efficiency. Based on this table, the *C*_*dl*_ value has increased in the solution with 800 ppm FV extract compared to 1000 ppm one, revealing a reduction in double-layer thickness and, as a result, a reduction in the thickness of adsorbed corrosion inhibitor on the surface. The reduction of the adsorbed layer thickness might be a symptom of the inhomogeneous adsorption of corrosion inhibitors on the MS surface. The following approach can be considered to analyze this behavior; Due to the concentration gradient, corrosion inhibitors tend to be adsorbed onto the metal surfaces when introduced to acid solutions. The adsorption process is optimized when the corrosion inhibitor molecules have the least interaction with each other and the most interaction with the metal surface. It is evident that elevating the concentration over the optimal concentration considerably increases the intermolecular attraction leading to the creation of clusters^50^. Therefore, a passageway might be created for the penetration of corrosive ions (Fig. 4). In fact, the inhibitor's intermolecular interaction at high concentrations to form clusters is more thermodynamically favored, which competes with adsorption forces between the inhibitor and the metal surface preventing them from developing a compact monolayer.

#### Polarization

**Figure 5:**
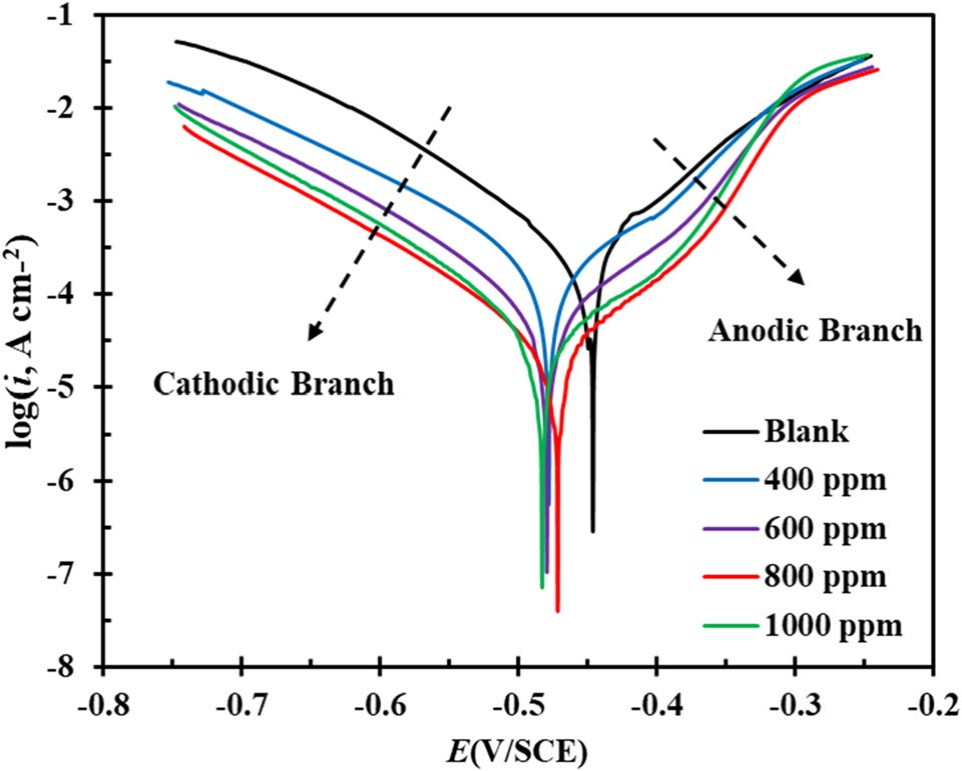
Polarization curves of MS submerged in blank and inhibitor-containing solutions.

Further investigation on the effect of FV leaves extract on cathodic and anodic reactions was investigated using polarization analysis after 24 h of submersion of MS in the electrolyte without and with various concentrations of the extract. Polarization parameters, comprising corrosion current density (*i*_*corr*_), corrosion potential (*E*_*corr*_) and slope of anodic and cathodic branches (*β*_*a*_ and *β*_*c*_), are listed in Table 2. According to Fig. 5, it can be realized that in the presence of FV leaves extracts, the current density has decreased in both anodic and cathodic branches compared to the blank. The increase in current density in the anodic branch at -0.3 V/SCE indicates the desorption of corrosion inhibitor molecules from the MS surface at high potential^51^. This potential is known as the desorption potential. The FV extract seems to have a more significant impact on the anodic reaction mechanism, as observed through changes in the anodic slopes. Meanwhile, the cathodic branch diagrams remain parallel in form, indicating that the cathodic reaction mechanism remains unchanged. The corrosion inhibition efficiency (ξ) can be calculated using Eq. (5), where *i*_*0*_ and *i*_*i*_ represent the MS corrosion current density in the blank and inhibitor-containing solution, respectively.

**Table 2:** Parameters acquired from polarization analysis.

	*βa* (mV/dec)	*−βc* (mV/dec)	*i*_*0*_ (µA/cm^2^)	*E*_*0*_ (V)	$\xi$ (%)	*θ*_*PDP*_
Blank	80.5	109.0	264.0	−0.446	–	–
400 ppm	109.3	116.2	185.1	−0.478	29.8	0.298
600 ppm	93.5	100.4	57.7	−0.479	78.1	0.781
800 ppm	84.5	103.8	20.4	−0.471	92.2	0.922
1000 ppm	99.7	95.1	33.5	−0.483	87.3	0.873

5 $$\xi \%=\left(1-\frac{{i}_{i}}{{i}_{0}}\right) 100\%$$

According to Table 2, the corrosion current densities' values have decreased with the addition of corrosion inhibitor, reaching 20.4 µA/cm^2^ at the optimum concentration (800 ppm), which is significantly lower than the blank (264.0 µA/cm^2^). Moreover, the inhibition efficiency at this concentration (800 ppm) is 92.2 %, highlighting this inhibitor's excellent ability to protect the MS against harsh species. Besides, since the difference in *E*_*corr*_ compared to blank is less than 85 mV, it can be concluded that FV extract acts as a mixed corrosion inhibitor^52^.

#### Adsorption* isotherms*

**Figure 6:**
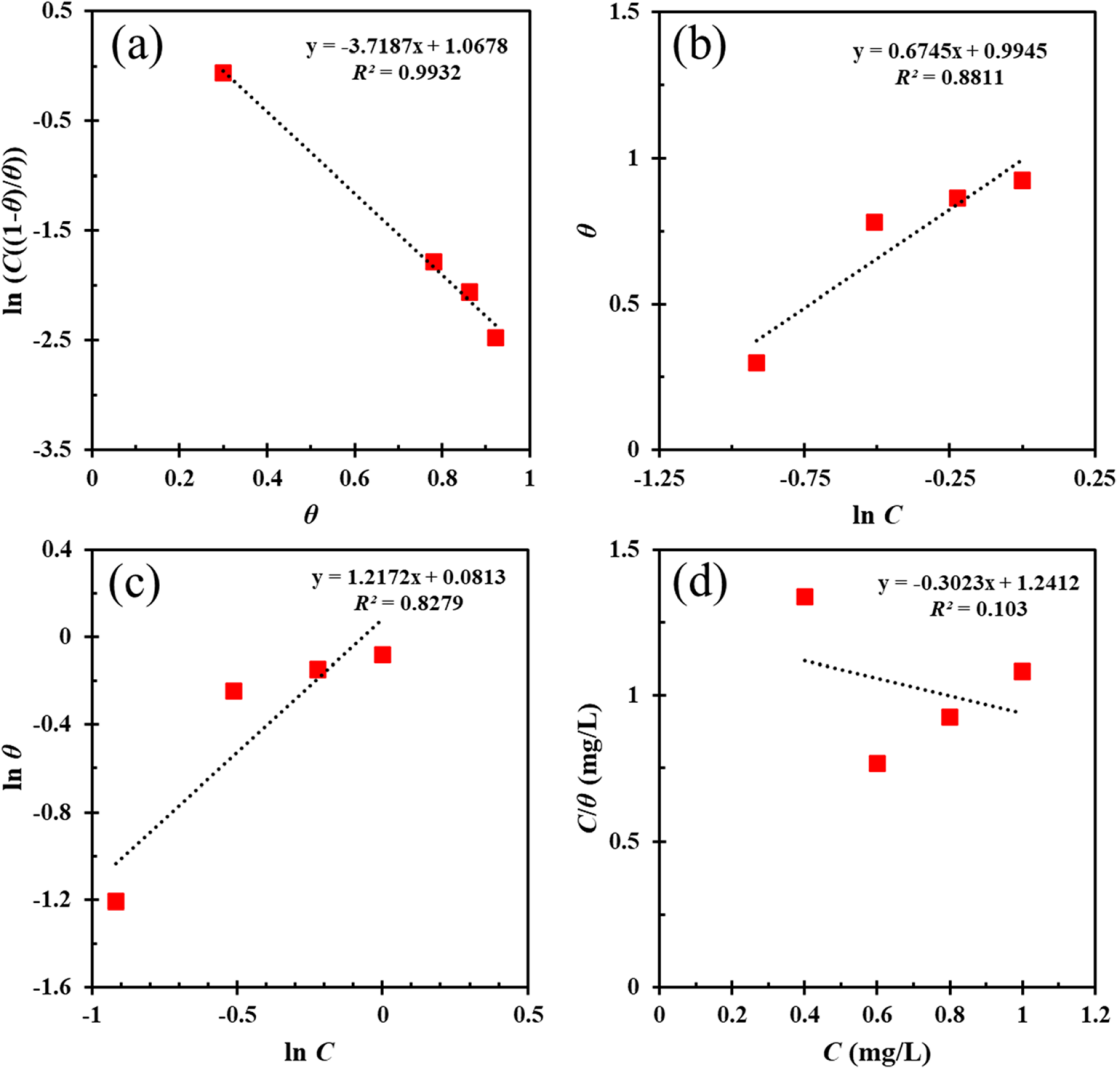
Frumkin (**a**), Temkin (**b**), Freundlich (**c**), and Langmuir (**d**) adsorption isotherms diagrams.

In order to explore the interaction of FV molecules and MS surface, different adsorption isotherms comprising Frumkin, Temkin, Freundlich and Langmuir were investigated. The adsorption isotherm equations are given below (Eqs. 6–9), and the corresponding diagram is shown in Fig. 6:

6 $$\mathrm{Frumkin}:\mathrm{ ln}\frac{C(1-{\theta }_{Pol})}{{\theta }_{Pol}}= -2\alpha {\theta }_{Pol}-\mathrm{ln}{K}_{ads}$$

7 $$\mathrm{Temkin}: \mathit{exp}\left(-2\alpha {\theta }_{Pol}\right)={K}_{ads}C$$

8 $$\mathrm{Freundlich}: {\theta }_{Pol}={K}_{ads}{C}^{n}$$

9 $$\mathrm{Langmuir}: \frac{C}{{\theta }_{Pol}}=\frac{1}{{K}_{ads}}+C$$

${\theta }_{Pol}(=\frac{\xi \%}{100})$, *α*, and *K*_*ads*_ in the above equations symbolize surface coverage based on polarization data, lateral interaction between FV leaves extract and MS surface, and adsorption/desorption equilibrium constant, respectively. According to Fig. 6, the best fit among several adsorption isotherms is the Frumkin isotherm with *R*^*2*^ = 0.9932. The values of *K*_*ads*_ and *α* derived from the intercept and slope of the Frumkin isotherm curve are shown in Table 3.

**Table 3:** Valuable parameters extracted from Frumkin isotherm.

Parameters	Value
*K*_*ads*_ (L/g)	0.345
*α*	1.86

**Figure 7:**
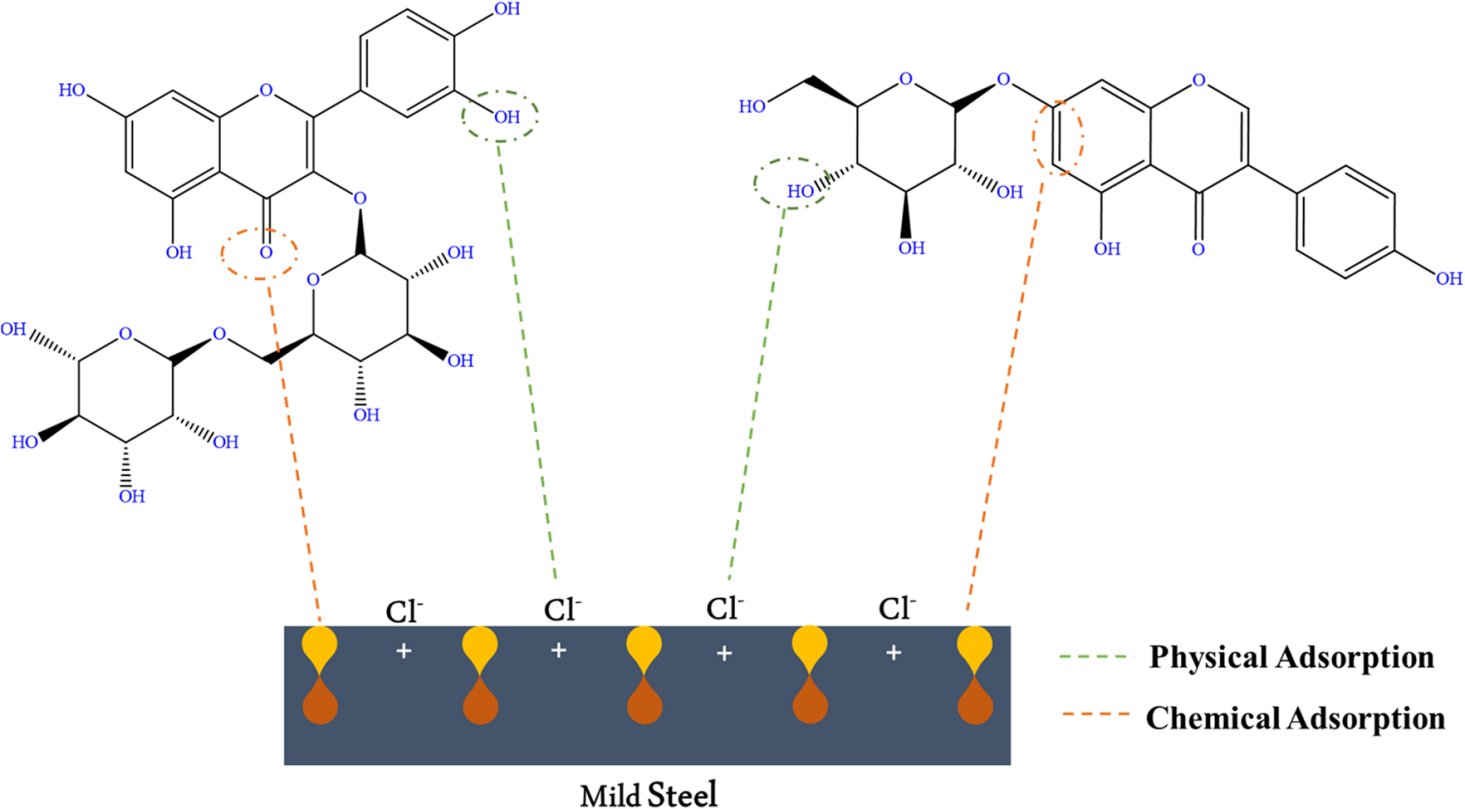
Schematic of the proposed mechanism of adsorption of FV leaves extract on MS surface.

The protective effects of natural extracts are believed to come from the presence of multiple molecules at different contents. One key factor is the synergistic interaction between these components, which increases their inhibiting effect^5,7,11^. The high concentration of compounds with aromatic rings containing heteroatoms (nitrogen, oxygen, and sulfur) enhances their ability to adsorb onto the metal surface and thus provides protection against corrosive ions such as H^+^ or Cl^−^. The possible adsorption mechanism of FV leaves extract molecules on the MS surface is shown in Fig. 7.

### Surface studies

#### FE-SEM/EDX

**Figure 8:**
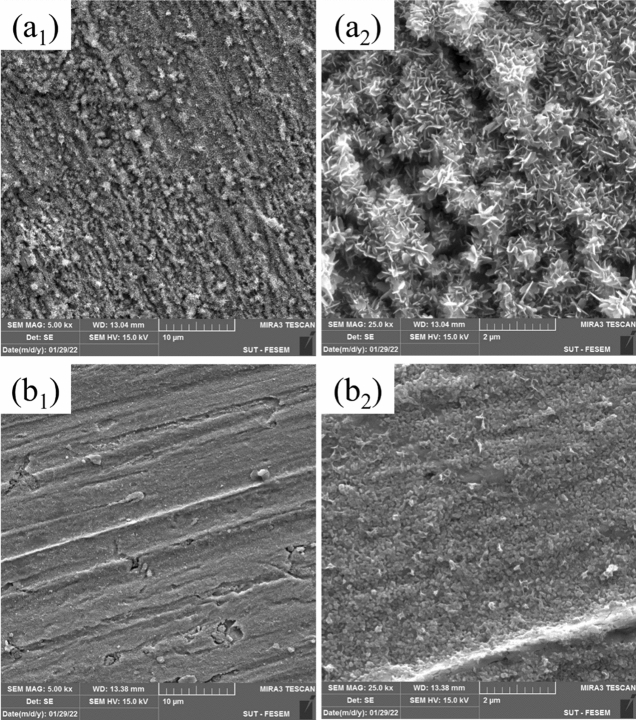
FE-SEM images of MS surface soaked in acidic solution without (**a**_**1**_**, a**_**2**_) and with 800 ppm FV extract (**b**_**1**_**, b**_**2**_).

The surface morphology of MS was investigated after submerging an MS coupon in the acidic solution in the absence and presence of 800 ppm FV extract for 6 h. As shown in FE-SEM images, the MS surface, which was exposed to the corrosive solution without inhibitor, is severely damaged (Fig. 8a1,a2); However, adding the FV extract to the acidic solution prevented the formation of corrosion products and a relatively uniform and smooth surface can be seen (Fig. 8b1,b2). Lower damage to the metal surface in the presence of FV extract could be due to the efficient adsorption of corrosion inhibitors, which diminish the contact between the corrosive ions and the MS surface and lessens the dissolution rate^53,54^.

EDX analysis was also performed to demonstrate the elemental composition of the MS surface, which can be seen in Table 4. According to this table, Fe, C, and O elements were detected on the surface of MS. When the FV extract was added to the acidic solution, the percentage of the carbon was increased on the metal surface, confirming the FV-based protective film's creation. Also, the decrease in oxygen percentage on the surface can be due to the reduction of corrosion products^29,55^.

**Table 4:** Elemental composition of MS surface immersed in acid media and the acidic solution containing 800 ppm FV extract for 6 h.

Weight %			Sample
O	C	Fe	
5.4	6.6	88.0	Blank
2.5	7.0	90.5	800 ppm

#### AFM

**Figure 9:**
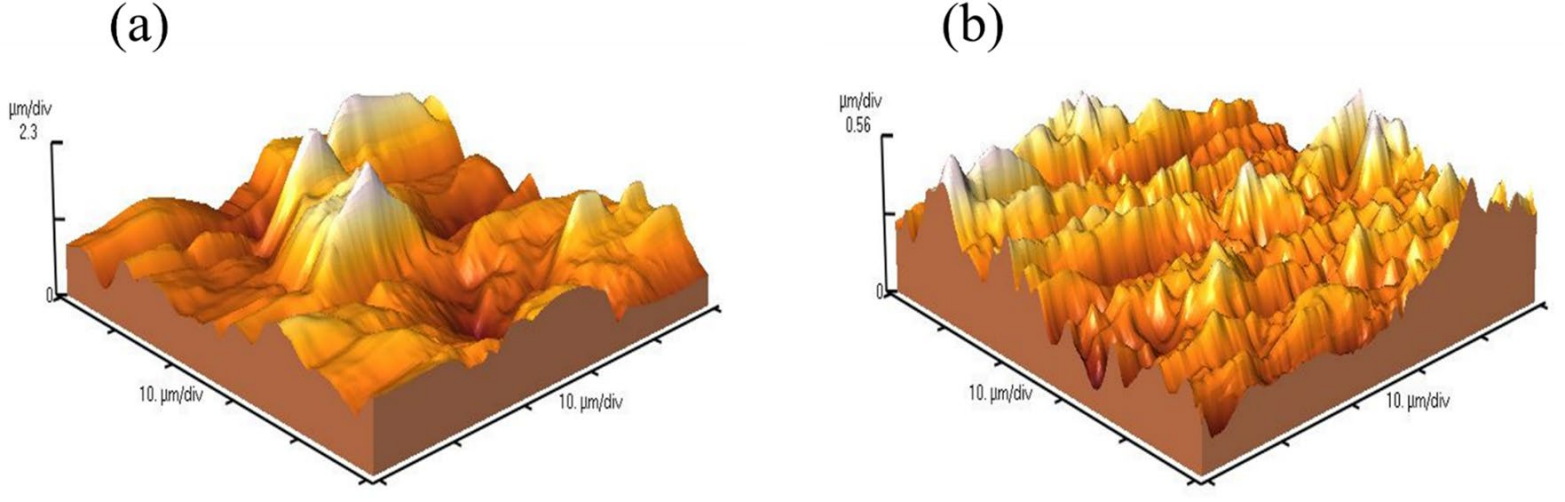
AFM micrograph of MS surface in 1 M HCl without (**a**) and with 800 ppm FV extract (**b**) after 6 h of immersion.

AFM analysis was conducted to demonstrate the effect of FV extract on the roughness and microstructure of the MS surface, which was submerged in 1 M HCl solution (Fig. 9). Based on the AFM results, the average roughness (*R*_*a*_) of the MS coupons in the blank sample was 520.4 nm, while in the presence of FV extract, this value decreased to 111.2 nm suggesting a decline in metal corrosion. Besides the *R*_*a*_, other parameters such as average height (*H*_*m*_), peak to valley (*R*_*p-v*_), and root mean square deviation (*R*_*q*_) are reported in Table 5. The reduction of these parameters establishes that the FV extract molecules adsorbed on the steel surface limited corrosion attacks on the MS surface^56,57^.

**Table 5:** AFM parameters of MS surface dipped in 1 M HCl solution in the presence and absence of 800 ppm FV extract.

Samples	Parameters			
	*R*_*a*_ (nm)	*R*_*q*_ (nm)	*R*_*p-v*_ (µm)	*H*_*m*_ (µm)
Blank	520.4	684.4	4.688	1.778
800 ppm	111.2	143.3	1.115	0.512

#### FTIR

**Figure 10:**
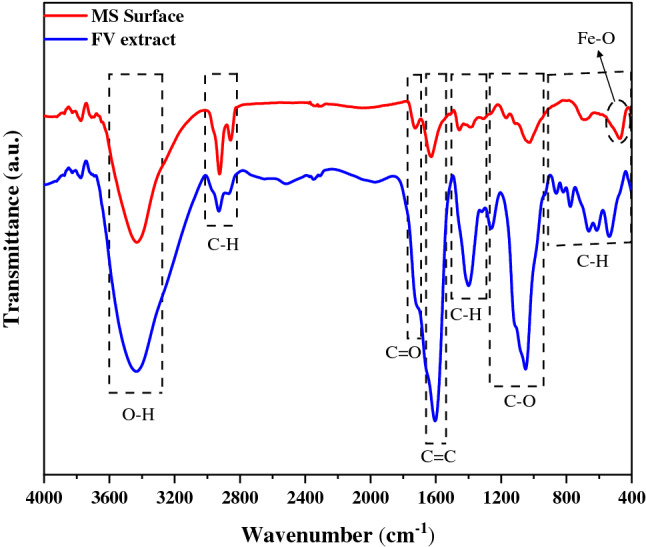
FTIR spectrum of the FV powder and MS immersed in the acidic solution containing 800 ppm FV extract after 6 h of immersion.

The FTIR spectrum was recorded to characterize the MS surface after immersing in an acidic solution containing FV extract. According to the FTIR spectrum of FV extract, which can be seen in Fig. 10, the peak at 3434 cm^−1^ is related to O–H stretching^58^. Moreover, the appearance of peaks at 2856 cm^−1^ and 2930 cm^−1^ are linked to the aliphatic CH_2_ stretching^43,58^. The peaks related to C=C stretching of the aromatic ring and C=O stretching are observed at 1605 cm^−1^ and 1715 cm^−1^, respectively^58,59^. The peaks between 1295 and 1490 cm^−1^ are correlated to C-H bending^59^. Besides, the peaks at 1056 cm^−1^ and 1260 cm^−1^ are linked to C–O and C–O–C stretching^60^. The peaks between 400 and 1000 cm^−1^ can be due to the aliphatic and aromatic C-H bending^30^. The presence of the FV extract peaks in the FTIR spectrum of the MS surface reveals that the adsorption of FV extract molecules on the metal surface occurs during immersion.

Additionally, the appearance of a new peak at 476 cm^−1^ is connected to the Fe–O bonds, revealing the interaction of the oxygen-containing groups of FV extract molecules and metal surfaces^61^. Furthermore, shifting the peaks of C=O stretching from 1715 to 1728 cm^−1^ and C=C stretching from 1605 to 1624 cm^−1^ can be due to the development of a complex between FV extract and iron ions ^21^. The results showed that functional groups, aromatic rings, and oxygen-containing groups in the FV extract structure could cause the interaction between FV extract compounds and the MS surface.

#### UV–Vis

**Figure 11:**
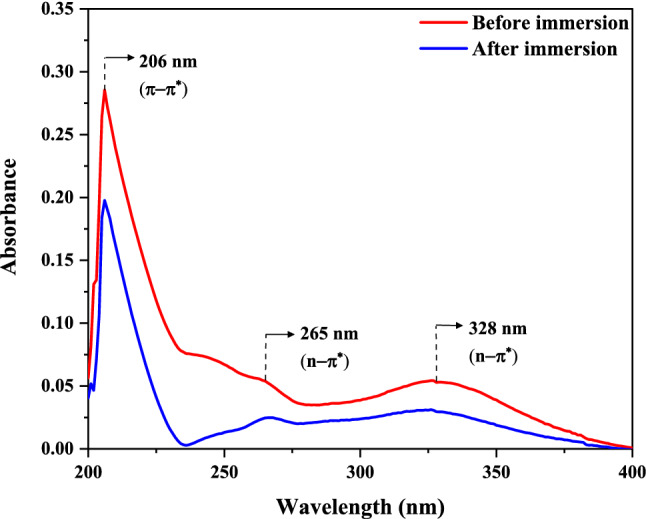
UV–Vis spectra of 1 M HCl solution containing 800 ppm FV extract before and after MS immersion.

The possibility of complex development among the FV extract compounds and iron ions was studied using the UV–Vis test. Figure 11 shows the UV–Vis spectrum before and after soaking the MS in the acidic solution containing FV extract. The UV–Vis spectrum of the solution before immersion, included three peaks centered at 206 nm, 265 nm, and 328 nm. Intensive absorption at 206 nm is related to π–π^*^ transitions of C=C bonds in aromatic rings, and the absorption at 265 nm and 328 nm are associated with n-π^*^ transitions of C=O and O–H bonds, respectively^61,62^. After immersion of the MS in the acidic solution containing the FV extract, the intensity of the absorption peaks related to π–π^*^ and n–π^*^ transitions dramatically decreased, and the UV–Vis spectrum shifted to a lower wavelength value (blue shift). These observations may correspond to the adsorption of FV extract molecules on the MS surface and the formation of an organic–inorganic complex via the interaction between pair electrons of FV extract molecule and vacant orbital of Fe^2+^/Fe^3+^, which causes the construction of a shielding film against corrosive species^63^.

#### GIXRD

**Figure 12:**
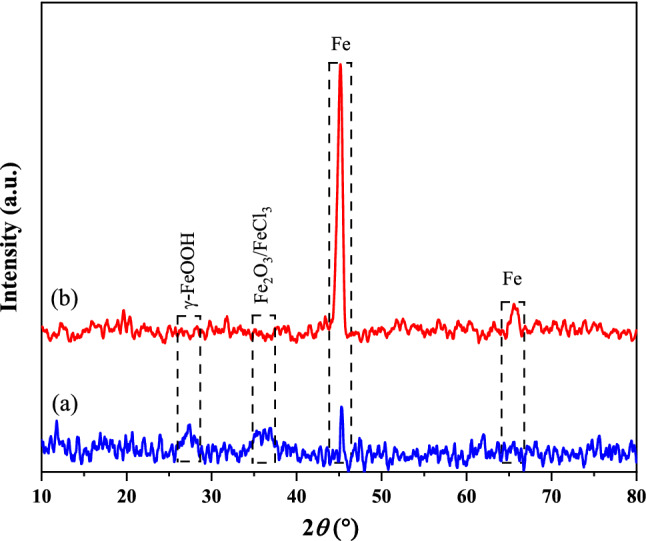
GIXRD patterns of the MS surface immersed in 1 M HCl (**a**) and 1 M HCl solution containing 800 ppm FV extract (**b**) after 6 h of immersion.

GIXRD was recorded to investigate the crystalline composition of the MS surface after soaking in an acidic solution in the presence and absence of FV extract. As shown in Fig. 12 the peak at 2*θ* = 27.3° is related to γ-FeOOH, and peaks at 2*θ* = 35.4°, 36.3°, and 36.9° are attributed to Fe_2_O_3_/FeCl_3_ which are due to the presence of corrosion product on the blank specimen surface. Also, peaks at 2*θ* = 45.2°, and 65.6° are related to iron metal^64,65^. Comparing the GIXRD patterns shows that the corrosion product peaks disappeared after adding FV extract to the acidic solution. Furthermore, the intensification of the peak at 45.2° and the appearance of a peak at 65.6° (peaks related to Fe metal) can confirm the assumption of the interaction between FV extract compounds and metal surface and verify the production of less corrosion products.

### Comparative study

Table 6 summarizes the corrosion inhibition capabilities of several plant extracts in terms of solvent utilized, optimal inhibitor concentration, inhibition efficiency at optimal concentration, and adsorption isotherm. According to this table, the FV leaves extract with an inhibition efficacy above 90% can be designated a robust MS corrosion inhibitor. Moreover, the extraction procedure is considered environmentally friendly because water was employed as the solvent.

**Table 6:** Comparison between different plant extract used as corrosion inhibitor for MS in HCl medium.

Plant extract	Extraction solvent	Optimum concentration (ppm)	Inhibition efficiency at the optimum concentration (%)	Adsorption isotherm	Ref
Soybean	Water	300	62	Langmuir	^66^
*Rheum ribes* root	Water	2000	85.9	Langmuir	^67^
*Origanum compactum*	Ethanol	400	90	Langmuir	^68^
Golpar leaves	Water	1000	91	Langmuir	^69^
*Garcinia livingstonei* leaves	Ethanol	4000	96.84	Langmuir	^70^
Cabbage	Water	100	95.66	Langmuir	^2^
Pomelo peel	Ethanol	8000	84.07	Langmuir	^71^
*Arbutus unedo* L. leaves	Ethanol/water	500	91.72	Langmuir	^30^
*Aerva lanata* flowers	Water	600	95.07	Langmuir	^72^
*Ceratonia siliqua* L seeds	Chloroform	100	93.84	Langmuir	^51^
*Thevetia peruviana* flower	Acetone/water	200	91.69	Langmuir	^73^
*Dolichandra unguis-cati* leaves	Ethanol	760	93.33	Langmuir	^74^
*Falcaria vulgaris* leaves	Water	800	91.3	Frumkin	Present study

## Conclusion

The current study sought to explore the application of FV extract as a sustainable and effective corrosion inhibitor for MS in hydrochloric acid. The results from EIS and polarization studies indicated that at an optimized concentration of FV (800 ppm), the inhibitor demonstrated a remarkable polarization resistance of 988.1 Ω cm^2^ and a 91.3% inhibition efficiency after 6 h of immersion. Additionally, there was a substantial reduction in corrosion current density by 92.2% compared to the blank sample. Surface coverage and inhibition efficiency data analyses revealed that the inhibitor was adsorbed horizontally on the metal surface, following the Frumkin adsorption isotherm. Furthermore, SEM and AFM analysis showed a smoother metal surface with fewer corrosion products, while EDX and FTIR verified the formation of an FV-based layer on the metal surface. In conclusion, these results demonstrate the potential of FV extract as an eco-friendly and efficient corrosion inhibitor for MS in hydrochloric acid media.

## Data Availability

All data generated or analysed during this study are included in this publish ed article.
